# Baicalein inhibits mitochondrial apoptosis induced by oxidative stress in cardiomyocytes by stabilizing MARCH5 expression

**DOI:** 10.1111/jcmm.14903

**Published:** 2019-12-27

**Authors:** Qi Li, Zhongjie Yu, Dandan Xiao, Yu Wang, Lin Zhao, Yi An, Yufang Gao

**Affiliations:** ^1^ Department of Emergency medicine The Affiliated Hospital of Qingdao University Qingdao China; ^2^ School of Medicine Qingdao University Qingdao China; ^3^ Institute for Translational Medicine Qingdao University Qingdao China; ^4^ School of Basic Medicine Qingdao University Qingdao China; ^5^ Nursing Department Office The Affiliated Hospital of Qingdao University Qingdao China; ^6^ Department of Cardiology The Affiliated Hospital of Qingdao University Qingdao China

**Keywords:** apoptosis, baicalein, cardioprotection, MARCH5, mitophagy, oxidative stress

## Abstract

Abnormal mitochondrial fission and mitophagy participate in the pathogenesis of many cardiovascular diseases. Baicalein is a key active component in the roots of traditional Chinese medicinal herb Scutellaria baicalensis Georgi. It has been reported that baicalein can resist cardiotoxicity induced by several stress, but the mechanisms of baicalein operate in the protection of cardiomyocytes need to be researched further. Here we report that baicalein can promote cell survival under oxidative stress by up‐regulating the expression level of MARCH5 in cardiomyocytes. Pre‐treatment cells or mice with baicalein can stabilize the expression of MARCH5, which plays a crucial role in the regulation of mitochondrial network and mitophagy. Overexpressed MARCH5 is able to against H_2_O_2_ and ischaemia/reperfusion (I/R) stress by suppressing mitochondrial fission and enhancing mitophagy, and then attenuate cells apoptosis. Altogether, our present study investigated that baicalein exerts a protective effect through regulating KLF4‐MARCH5‐Drp1 pathway, our research also provided a novel theoretical basis for the clinical application of baicalein.

## 1 INTRODUCTION

Mitochondrial homoeostasis is required for normal cell physiology,^1^ abnormal mitochondrial dynamics and mitophagy participate in the regulation of pathogenesis of heart diseases.^2^, ^3^, ^4^, ^5^ Excessive mitochondrial fission promotes cell apoptosis; inversely, mitochondrial fusion is able to inhibit apoptosis,^1^ while growing number of evidence reveals that unbalanced mitophagy also can induce cell apoptosis.^3^, ^6^, ^7^

The membrane‐associated RING‐CH (MARCH) proteins belong to the RING finger protein family of E3 ubiquitin ligases. MARCH5, one of MARCH protein family, is mainly localized on the mitochondrial outer membrane. MARCH5 has been considered to play roles in immune regulation, protein quality control and membrane transport by ubiquitinating different proteins.^8^, ^9^ Recent findings advocate that MARCH5 plays a critical role in regulating mitochondrial morphology and apoptosis.^10^ Loss of MARCH5 enhances apoptosis and promotes mitochondrial fission in HEK293, HeLa cells^11^ and HCT116 cells.^12^ Abundantly expressed MARCH5 in COS7 cells promotes the formation of long tubular mitochondria^13^ and BC cells growth.^14^ Mitophagy is responsible for eliminating of damaged mitochondria and regulating apoptosis in HeLa cells, which is also regulated by the MARCH5 level.^15^ However, it is not yet clear whether MARCH5 participates in the regulation of mitochondrial dynamics in cardiomyocytes and how mitophagy links with the mitochondrial fission.

Baicalein (5,6,7‐trihydroxyflavone) is one of the major phenolic flavonoids extracted from the root of Scutellaria baicalensis Georgi,^16^, ^17^ which has been widely applied in traditional Chinese medicine. It has been reported that baicalein has the effects of anti‐inflammatory, antioxidant and anti‐cancer.^18^, ^19^, ^20^ Recently, studies have shown that the antioxidant activities of baicalein can inhibit lung mitochondrial lipid peroxidation during ROS stress^21^ and decrease myocardial tissue injury undergo I/R in rats. However, whether baicalein is involved in the regulation of mitochondrial dynamics and mitophagy need further in‐depth studies.

Our present work reveals that MARCH5 plays a key role in regulating mitochondrial dynamics and mitophagy. Overexpression MARCH5 could promote mitochondrial fusion, mitophagy, and decreases cell apoptosis. Loss of MARCH5 increased mitochondrial fission, blocked mitophagy and promotes cell apoptosis. Baicalein could attenuate oxidative damage by stabilizing MARCH5 level in cell and tissue. We also revealed that baicalein played a role in protecting cell and tissue via regulating KLF4‐MARCH5‐Drp1 pathway. Our data shed new light on the understanding of MARCH5 in molecular regulation of mitochondrial network and laid a theoretical foundation of baicalein in clinical application.

## 2 METHODS

### 2.1 Cell cultures and treatment

H9C2 cells were cultured in DMEM (Gibco) with 10% foetal bovine serum (TransGen), and 100 U/mL penicillin, 100 mg/mL streptomycin (Invitrogen) in a humidified 5% CO_2_ incubator at 37°C. When the cultured cells reached approximately 70% confluently, they were treated with H_2_O_2_ (100 μM) incubated at 37°C for 3‐24 hours in complete culture medium. For tested the protective effect of baicalein (Invitrogen), we pre‐treated cells with baicalein (50 μM) for 4 hours and then incubated with H_2_O_2_ as above described.

### 2.2 The MARCH5 plasmid construction

The expression plasmids for MARCH5 was generated by amplifying the corresponding cDNA by PCR with Phanta Max Super‐Fidelity DNA Polymerase (Vazyme), and then cloning it into pcDNA3.1 expression vector by using ClonExpress Ultra One Step Cloning Kit (Vazyme). We used Lipofectamine 3000 (Thermo Fisher) for transfection vector. The procedures were in accordance with the kit instructions.

### 2.3 RNA interference assay

The MARCH5 siRNA was synthesized in BGI. The sequence of MARCH5 interference RNA is 5’‐GGUGCAGAGGAUCUACUAATT‐3’. We used Lipofectamine 3000 (Thermo Fisher) for transfection siRNA. The procedures were in accordance with the kit instructions.

### 2.4 Mitochondrial staining and analysis of mitochondrial fission

Mitochondrial staining was performed as others described with modifications.^1^, ^22^ Briefly, cells were plated onto the poly‐L‐lysine coated coverslips. After treatment, they were stained for 30 minutes with 0.02 µM MitoTracker Red at 37°C. Mitochondria were imaged using a laser‐scanning confocal microscope (Zeiss LSM510 META).

### 2.5 Apoptosis assays

Apoptosis was determined by the terminal deoxynucleotidyl transferase‐mediated dUTP nick‐end‐labelling (TUNEL) using a kit from TransGen. The detection procedures were in accordance with the kit instructions.

### 2.6 Immunoblotting

Immunoblot was carried out as others described.^23^ Briefly, the cells were lysed for 20 minutes on ice in RIPA lysis buffer containing a protease inhibitor cocktail and DMSF. The samples were subjected to 12% SDS‐PAGE and transferred to nitrocellulose membranes. Blots were probed with primary antibodies anti‐MARCH5 (Abcom, 1:1000), anti‐Lc3 (Abclone, 1:1000) anti‐KLF4 (Affinity, 1:1000), anti‐Drp1 (Affinity, 1:1000), anti‐Ubiquitin (Affinity, 1:1000) and anti‐β‐actin (TransGen, 1:2000) at 4°C overnight with gently shaking. After three times washing with PBS, the horseradish peroxidase (HRP)‐conjugated secondary antibodies were added. Antigen‐antibody complexes were visualized by enhanced chemiluminescence. Enhanced ECL TM prime detection reagent (GE) used to visualize antigen‐antibody complexes, the density quantified by Image J.

### 2.7 Immunoprecipitation

Cells were lysed on ice for 20 minutres in 500 μL NP‐40 lysis buffer (50 mM Tris‐HCl pH 8.0, 150 mM NaCl, 1 mM EDTA, 1% NP‐40, 10% glycerol, 0.2 mM PMSF and Protease inhibitor). The lysates were precleared by centrifugation, and 50 μL of the samples were aliquoted for input. The remaining samples were immunoprecipitated with 2 μg of antibody (MARCH5, Abcom; Drp1, Affinity) and 50 μL of Protein‐A/G PLUS‐Agarose (Santa Cruz Biotechnology). The samples were rotated at 4°C overnight. The beads were washed three times with 1 mL of low‐salt NP40 lysis buffer (300 mM NaCl) and twice with 1 mL of high‐salt lysis buffer (500 mM NaCl). The beads were then boiled for 10 minutes in the presence of 25 μL 2× sample buffer, and the released proteins were fractionated in 12% SDS‐PAGE gels. Proteins were detected by immunoblotting as described above.

### 2.8 Real‐time quantitative PCR

RT‐qPCR for MARCH5 was performed on a CFX96 Real‐Time PCR Detection System (Bio‐Rad). Total RNA was extracted using Trizol reagent. Reverse transcription reactions were carried out using the TransScript II One‐Step gDNA Removal and cDNA Synthesis SuperMix (TransGen) to make cDNA according to the manufacturer's guide. TransStart Green qPCR SuperMix (TransGen) was used for quantitative PCR (qPCR) analysis, and the procedures were in accordance with the kit instructions. The levels of MARCH5 analysed by RT‐qPCR were normalized to that of GAPDH. MARCH5 primers were forward: 5′‐ ATGCCGGACCAAGCCCTT‐3′ and reverse: 5′‐ TTATGCTTCTTCTTGCTCTGGATAATTTAGGAT‐3′. GAPDH forward primer: 5′‐ GTCGTGGAGTCTACTGGCGTCTTCA‐3′ and reverse: 5′‐ TCGTGGTTCACACCCATCACAAACA‐3′.

### 2.9 Autophagic flux

Autophagic flux experiment was performed as others described with some modifications.^24^ Autophagy flux was assessed with transduced Ad‐RFP‐GFP tandem‐tagged LC3. RFP retains its fluorescence even in the acidic environment of lysosomes where GFP loses its fluorescence. Thus, green LC3 puncta primarily indicate autophagosomes, while red LC3 puncta indicate both autophagosomes and autolysosomes. The red puncta that overlay with the green ones and appear yellow in merged images are indicators of autophagosomes, while the free red puncta that do not overlay with the green ones and appear red in merged images are indicative of autolysosomes. The puncta were imaged using a laser‐scanning confocal microscope (Zeiss LSM510 META).

### 2.10 Animal experiments

Male adult C57BL/6 mice (8 weeks old) were obtained from Qingdao Daren Fortune Animal Technology Co., Ltd. All experiments were performed according to the protocols approved by the Animal Care Committee, The Affiliated Hospital of Qingdao University.

We performed the I/R injury model as previously described with some modifications,^1^, ^22^

Male C57 mice, 8‐week‐old, were randomly divided into four groups: the sham group (sham, n = 5); the I/R control group (I/R, n = 5); the DMSO and I/R treated group (I/R + DMSO n = 5); the baicalein (25 mg/kg) and I/R treated group (I/R + BAI, n = 5), received an intraperitoneal injection of 40 mg/kg baicalein dissolved in DMSO. And mice were subjected to 30 minutes of left anterior descending coronary artery (LAD) ligation followed by 3 hours of reperfusion. The heart was rapidly excised. The heart slices were used for cardiomyocyte apoptosis analysed.

### 2.11 Statistical analysis

The results are expressed as mean ± SEM of at least three independent experiments. The statistical comparison among different groups was performed by one‐way analysis of variance (ANOVA) for multiple comparisons. Statistical analyses were performed with GraphPad Prism 5.0 (GraphPad Software, Inc, San Diego, CA). *P* < .05 was considered statistically significant.

## 3 RESULTS

### 3.1 H_2_O_2_ is able to induce mitochondrial fission, apoptosis and change mitophagy flux

**Figure 1:**
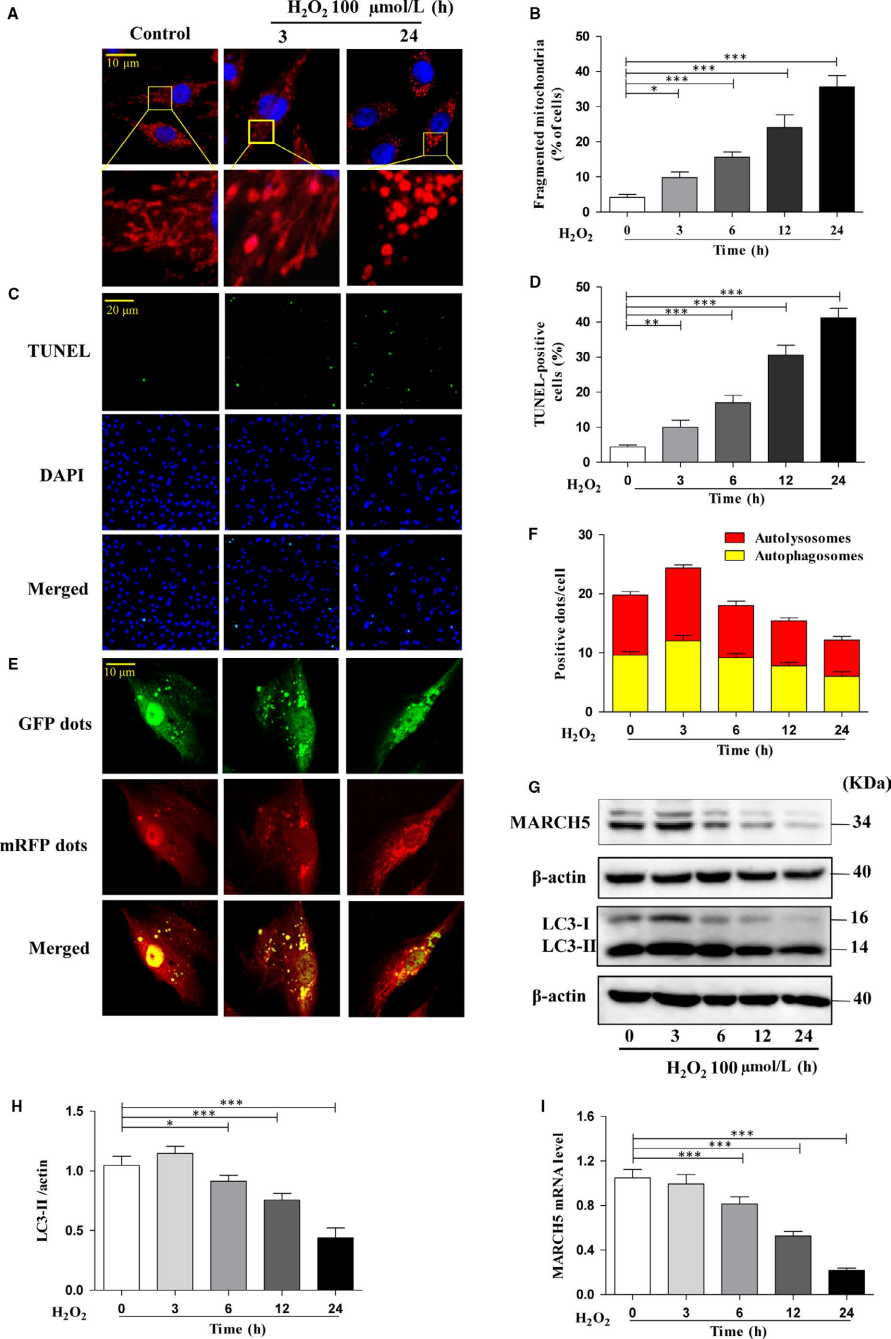
H_2_O_2_ exposure induces cardiotoxicity. H9C2 cells were exposed to 100 μm H_2_O_2_ for the indicated time. (A) Mitochondrial morphology was stained with MitoTracker Red and observed using a laser‐scanning confocal microscope. (B) shown the percentage of cells undergoing mitochondrial fission. (C) Apoptotic cells were detected by TUNEL assay and the percentage of apoptotic cells shown in (D). (E) Autophagy flux was assessed with transduced Ad‐RFP‐GFP tandem‐tagged LC3. Autophagy flux was observed using a laser‐scanning confocal microscope the numbers of autolysosomes and autophagosomes in H9C2 cells (F). mRFP dots (red) indicated autolysosomes, and the merged (yellow) dots indicated autophagosomes. The expression level of MARCH5 was detected by Western blotting (G) and RT‐qPCR (I). LC3 protein expression level was detected by Western blotting (G), densitometry (H). All of the data were expressed as the mean ± SEM of three independent experiments. *P < .05, **P < .01, ***P < .001

To explore whether MARCH5 is involved in the regulation of oxidative stress‐induced mitochondrial fission, mitophagy and apoptosis in cardiomyocyte, we treated the H9C2 cells with H_2_O_2_ and determined the change of cell dynamics at different time points. We observed a time‐dependent increase in the mitochondrial fission (Figure 1A,B). Abnormal mitochondrial fusion and fission take part in the regulation of apoptosis, meanwhile we discovered an incremental tendency of TUNEL positive cells with prolonged exposure time (Figure 1C,D). Unlike mitochondrial fission and apoptosis, the mitophagy flux increases at the first stage (3 hours), and then decreases with the extension of exposure time(6 ~ 24 hours) (Figure 1E,F).

LC3 is widely used as autophagy marker and up‐regulated with autophagy occurrence.^25^, ^26^ We detected the expression level of LC3 in H9C2 cells treatment with H_2_O_2_ at different time points and found that the tendency of LC3 is similarly to mitophagy flux. The expression level of LC3‐II is little higher in 3 hours, and then lower upon H_2_O_2_ exposure time (Figure 1G,H). Since H_2_O_2_ can induce mitochondrial fission, suppress mitophagy and then enhance cell apoptosis, we further want to understand whether MARCH5 links with these events. We noted that the expression level of MARCH5 markedly decreases with the growth over time (Figure 1G,I). These data suggested that hydrogen peroxide could induce mitochondrial fission, inhibit mitophagy and a concomitant decrease in cell viability. Time‐dependent decreased of MARCH5 level revealed that it might tightly link with these cellular events.

### 3.2 Overexpression of MARCH5 reduces H_2_O_2_ induced cardiotoxicity

**Figure 2:**
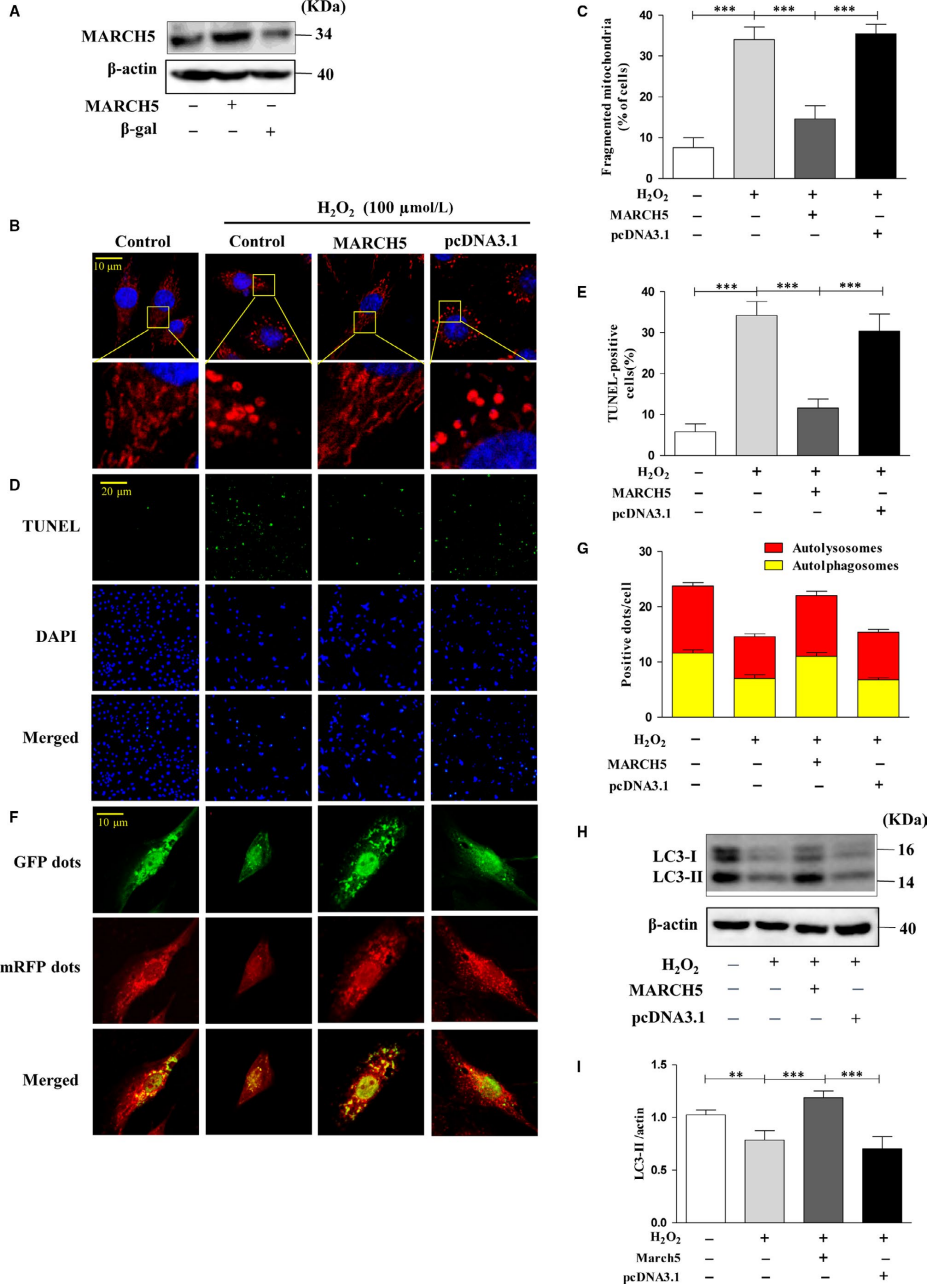
Overexpression of MARCH5 reduces cardiotoxicity induced H_2_O_2_. (A) H9C2 cells were transfected with MARCH5‐cDNA for 24 h, and the expression level of MARCH5 was detected by Western blotting. (B) H9C2 cells were exposed to 100 μM H_2_O_2_ for another 24 h, and mitochondrial morphology was stained with MitoTracker Red and observed using a laser‐scanning confocal microscope, (C) shown the percentage of cells undergoing mitochondrial fission. Apoptotic cells were detected by TUNEL assay (D) and the percentage of apoptotic cells shown in (E). Autophagy flux was assessed with transduced Ad‐RFP‐GFP tandem‐tagged LC3. Autophagy flux was observed using a laser‐scanning confocal microscope (F) and (G) shown the numbers of autolysosomes and autophagosomes. LC3 protein was detected by Western blotting (H) and densitometry (I). All of the data were expressed as the mean ± SEM of three independent experiments. *P < .05, **P < .01, ***P < .001

To systematically understanding the critical role of MARCH5 in H_2_O_2_ induced cardiotoxicity, we transfected the cells with the plasmid construct of MARCH5‐cDNA to induce MARCH5 overexpression. The expression level of MARCH5 was significantly increased by its plasmid but not by its control (Figure 2A). As observed in Figure 2B,C, the MARCH5 overexpressed cells show less mitochondrial fission as compared to negative and empty vector control. The cells were treated with H_2_O_2_ for 24 hours, and the percentage of mitochondrial fission, TUNEL and mitophagy efflux positive signal were counted for measure mitochondrial dynamics. The results showed overexpressed MARCH5 in cells can significantly against H_2_O_2_ induced cardiotoxicity via inhibition mitochondrial fission, promotion mitophagy (Figure 2F,G) and decrease apoptosis (Figure 2D,E) as compare with the negative and empty vector control. We also tested the expression level of LC3‐II and observed a significantly increased level of LC3‐II in MARCH5 overexpression group than the control groups (Figure 2H,I). These results discovered that overexpressed MARCH5 in cardiomyocytes can protect cells from cardiotoxicity induced by H_2_O_2_.

### 3.3 Knockdown of MARCH5 sensitizes H9C2 cells to H_2_O_2_ caused cardiotoxicity

**Figure 3:**
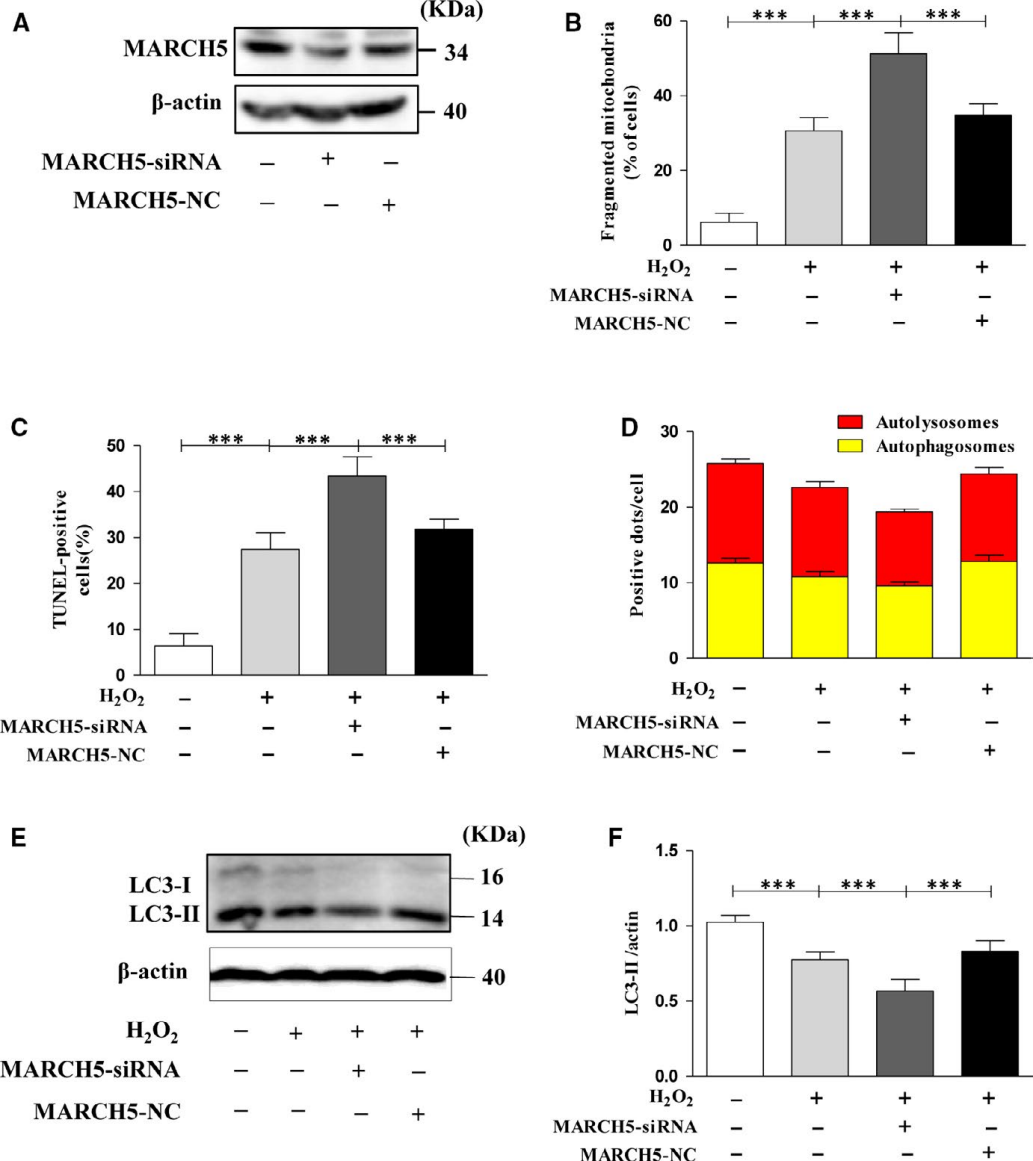
Knockdown of MARCH5 sensitizes H9C2 cells to H^2^O^2^ caused cardiotoxicity. H9C2 cells were transfected with MARCH5‐siRNA for 24 h, and the expression level of MARCH5 was detected by Western blotting (A). H9C2 cells were exposed to 50 μM H_2_O_2_ for another 24 h, and mitochondrial morphology was stained with MitoTracker Red and observed using a laser‐scanning confocal microscope, (B) shown the percentage of cells undergoing mitochondrial fission. Apoptotic cells were detected by TUNEL assay the percentage of apoptotic cells was shown in (C). Autophagy flux was assessed with transduced Ad‐RFP‐GFP tandem‐tagged LC3. (D) shown the numbers of autolysosomes and autophagosomes in H9C2 cells. LC3 protein was detected by Western blotting (E) and densitometry (F). All of the data were expressed as the mean ± SEM of three independent experiments. *P < .05, **P < .01, ***P < .001

To examine wether MARCH5 is the key factor of attenuating H_2_O_2_ caused cardiotoxicity, we knocked down the endogenous MARCH5 expression using MARCH5‐siRNA. The expression level of MARCH5 was significantly decreased by MARCH5‐siRNA but not by its control (Figure 3A). Lower dose of H_2_O_2_ (50 μM) was used for treatment cardiomyocytes, and we explored whether MARCH5 knockdown is able to enhance the sensitivity of cardiomyocytes to H_2_O_2_ induced cardiotoxicity. As shown in Figure 3B, the percentage of fragmented mitochondrial cells significantly higher in MARCH5 knockdown group than other groups. The MARCH5‐knockdown cells decreased the mitophagy sensitivity and increased the number of apoptosis cells (Figure 3C,D). The expression level of LC3‐II in MARCH5‐siRNA group was significantly reduced (Figure 3E,F), which further defined that loss MARCH5 could inhibit the mitophagy. These data revealed the importance of MARCH5 in resisting H_2_O_2_ induced cardiotoxicity, cells loss of MARCH5 was peculiarly susceptible to oxidative damage caused by H_2_O_2_ treatment.

### 3.4 Protective effect of baicalein on H_2_O_2_ induced cardiotoxicity

**Figure 4:**
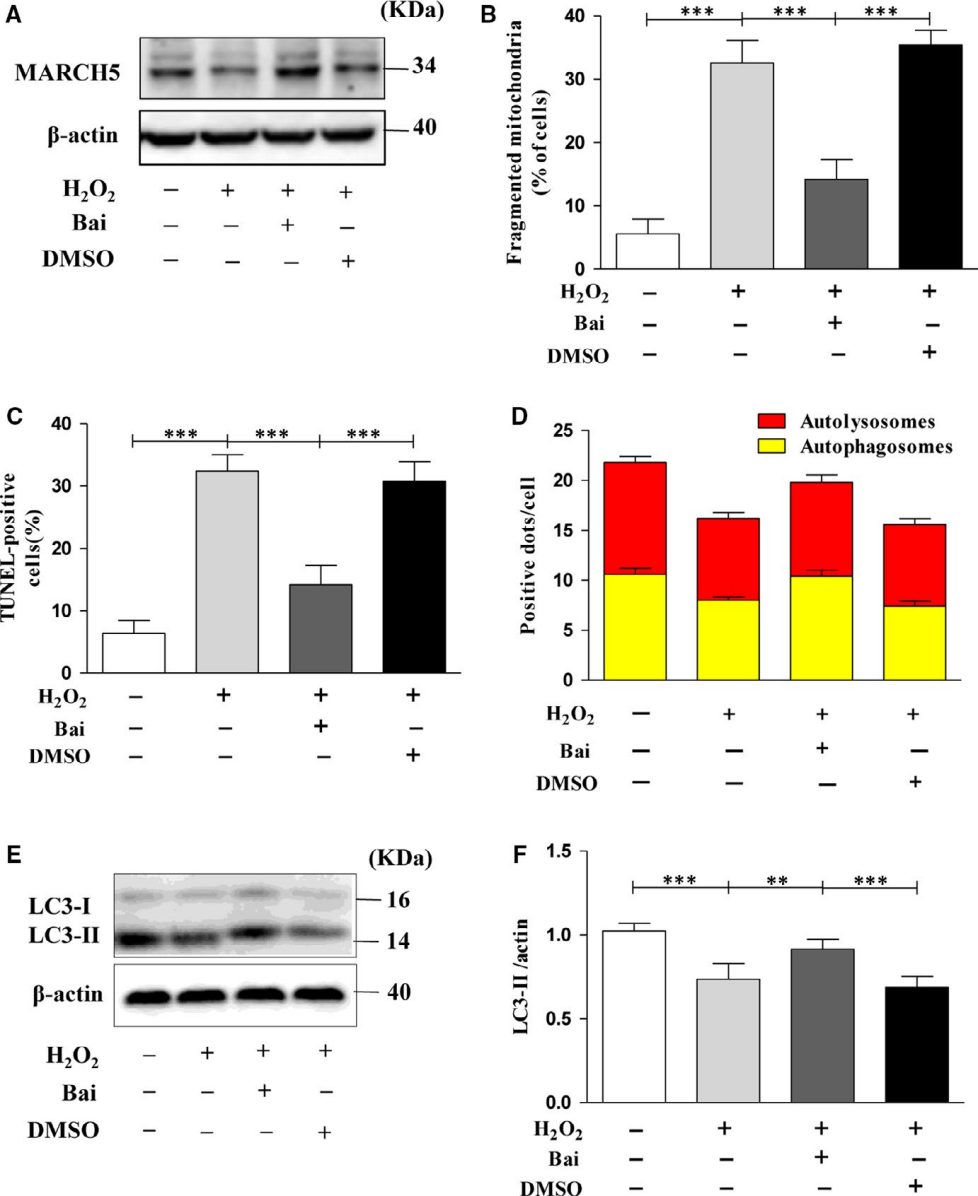
Protective effect of baicalein on H_2_O_2_ induced cardiotoxicity. H9C2 cells were pre‐treated with 50 μM baicalein for 4 h, then H9C2 cells were exposed to 100 μM H_2_O_2_ for 24 h, and the expression level of MARCH5 was detected by Western blotting (A). The percentage of cells undergoing mitochondrial fission is shown as (B). The percentage of apoptotic cells is shown as (C) detected by TUNEL assay. The numbers of autolysosomes and autophagosomes were observed as (D). LC3 protein was detected by Western blotting (E) and densitometry (F). All of the data were expressed as the mean ± SEM of three independent experiments. *P < .05, **P < .01, ***P < .001

Baicalein is a key active phenolic flavonoids in the roots of Scutellaria baicalensis Georgi,^16^ which has been reported to have multi‐functions. To determine whether baicalein protects the cardiomyocytes against oxidative stress injury, we pre‐treated H9C2 cells with baicalin for 4 hours before exposure under H_2_O_2_. Strikingly, the data showed that pre‐treated with baicalin can reverse the oxidative damage induced by H_2_O_2_. The number of cells with fragmented mitochondria was significantly reduced in baicalin group compared with other groups (Figure 4B). Baicalein also can promote cardiomyocytes mitophagy (Figure 4D–F) and inhibit apoptosis (Figure 4C). To determine whether baicalein play its role by regulation of MARCH5, we tested the expression level of MARCH5 and found that the level of MARCH5 increased significantly in baicalein group during the oxidative damage caused by H_2_O_2_ (Figure 4A). These results pointed out that baicalein can against H_2_O_2_ induced cardiotoxicity by stabilizing the expression of MARCH5.

### 3.5 The protective mechanism of baicalein on H_2_O_2_ induced cardiotoxicity

**Figure 5:**
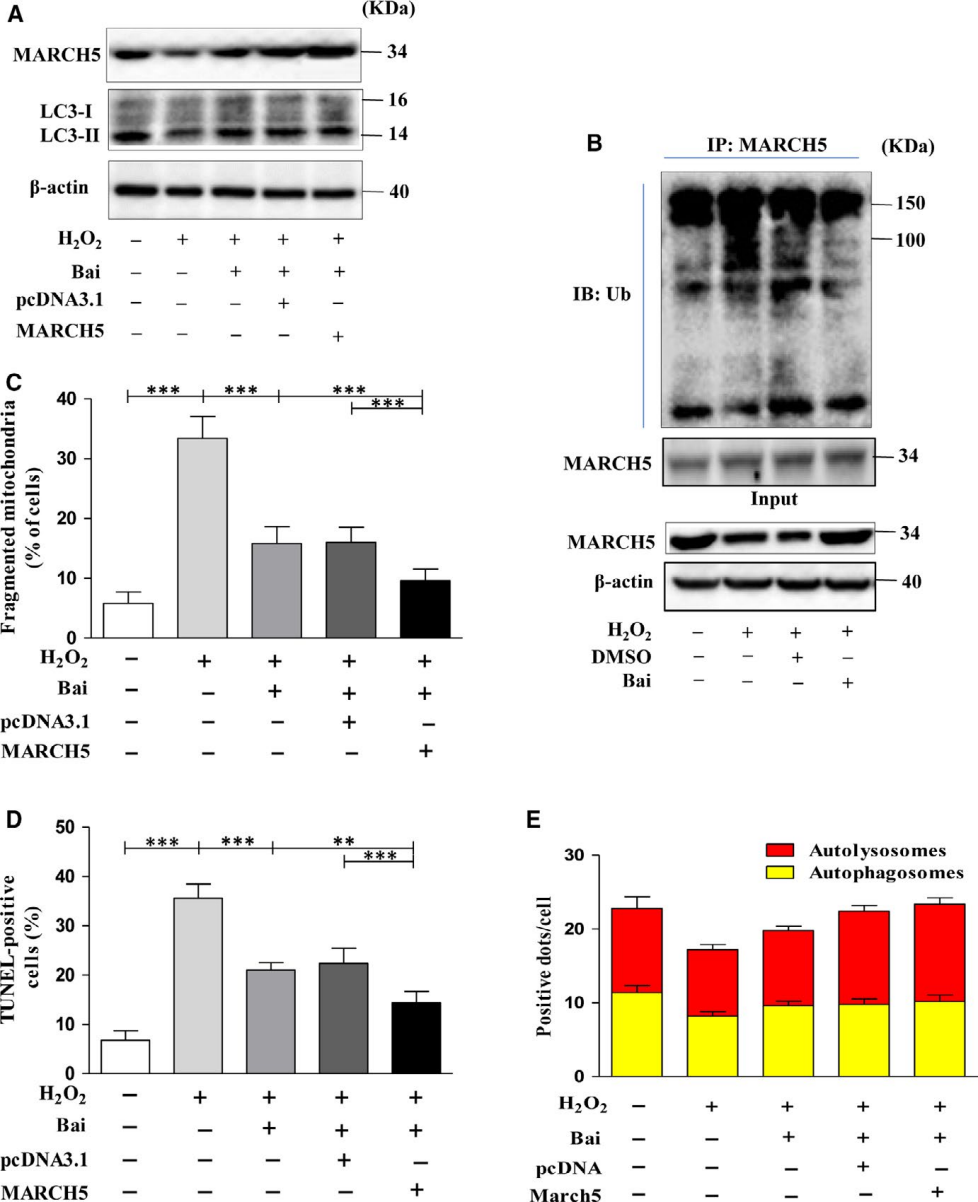
The protective mechanism of baicalein on H_2_O_2_ induced cardiotoxicity. H9C2 cells were exposed to 100 μM H_2_O_2_ for 24 h after pre‐treated with 50 μM baicalein for 4 h, transfected with MARCH5‐cDNA for 24 h, pre‐treated with baicalein and transfected with MARCH5‐cDNA, respectively. MARCH5 and LC3 proteins were detected by Western blotting. (A) Ubiquitylation assays were performed as described in methods. The ubiquitylation level of MARCH5 was detected using an Ub antibody (B). (C) shown the percentage of cells undergoing mitochondrial fission. (D) shown the percentage of apoptotic cells. (E) shown the autolysosomes and autophagosomes in H9C2 cells. All of the data were expressed as the mean ± SEM of three independent experiments. *P < .05, **P < .01, ***P < .001

To understand the underlying protective mechanism of baicalein, based on the results shown in Figure 4, we first treated the H9C2 cells with H_2_O_2_ after pre‐treatment with baicalein and transfection with MARCH5‐cDNA. We found that in baicalein pre‐treatment group and MARCH5 overexpression group, the number of mitochondrial fission cells was remarkably reduced (Figure 5C), mitophagy was enhanced (Figure 5A,E), and the number of apoptotic cells was significantly decreased (Figure 5D). These data revealed that baicalein had the similar protective function with overexpression MARCH5 via increasing the MARCH5 expression (Figure 5A). We also observed the synergetic protective effect of baicalein and MARCH5 on H_2_O_2_ induced cardiotoxicity (Figure 5C–E). Most recent studies have revealed that ubiquitination can promote apoptosis,^27^ we then tested the modification of MARCH5 by ubiquitin. As shown in Figure 5B, the mount of ubiquitin‐MARCH5 noticeable increased compared with control under oxidative stress. In baicalein groups, the ubiquitination level striking decreased as compared with its related control (Figure 5B). All the data illuminated that baicalein can attenuate the ubiquitination level of MARCH5 and consequently stabilize intracellular MARCH5 level. Stably expression of MARCH5 is essential for maintaining the homoeostasis of mitochondrial dynamics and mitophagy.

**Figure 6:**
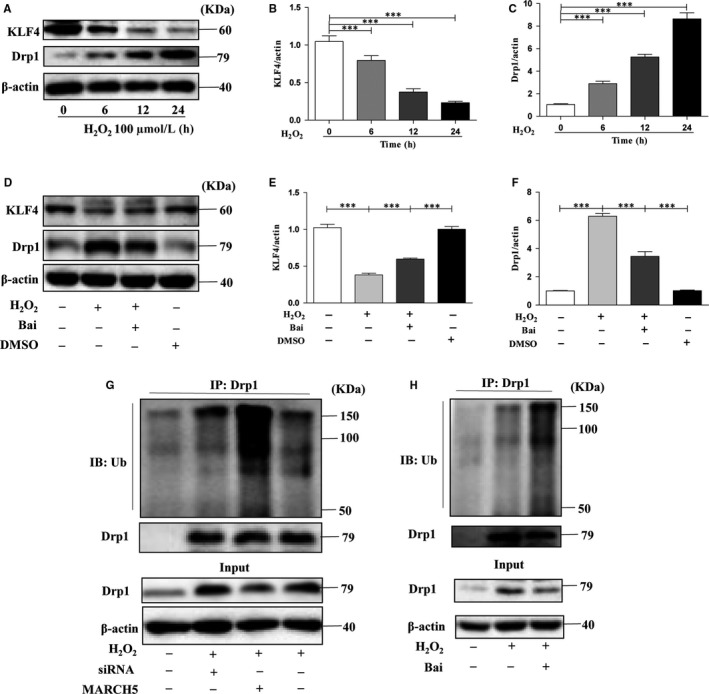
The cardioprotective pathway regulated by baicalein. H9C2 cells were exposed to 100μM H_2_O_2_ for 24 h after pre‐treated with 50 μM baicalein for 4 h, transfected with MARCH5‐cDNA or MARCH5‐siRNA for 24 h, respectively. KLF4 and Drp1 proteins were detected by Western blotting. (A) The expression level of KLF4 and Drp1 in different time groups after treatment with H2O2, (B, C) The densitometry of KLF4 and Drp1. (D) The expression level of KLF4 and Drp1 in different groups of pre‐treated with baicalein or not, (E, F) The densitometry of KLF4 and Drp1. (G) The ubiquitylation level of Drp1 in normal, MARCH5‐siRNA and MARCH5‐cDNA after treatment with H2O2. (H) The ubiquitylation level of Drp1 in normal and pre‐treated with baicalein after treatment with H2O2. All of the data were expressed as the mean ± SEM of three independent experiments. *P < .05, **P < .01, ***P < .001

To further investigate the protective effect of baicalein via MARCH4 pathway, we detected the expression level of KLF4, which is a transcriptional regulatory factor of MARCH5.^28^ As showed in Figure 6A,B, the expression level of KLF4 gradually decreased with extended time of response to H_2_O_2_, but in the baicalein treatment group, the expression level of KLF4 was remarkably increased when compared with H_2_O_2_ group (Figure 6D,E).The expression trend of KLF4 was similar with MARCH5 (Figures 1A and 5A), and these data suggested that treatment with H_2_O_2_ can significantly reduce the expression level of MARCH5 via inhibit the expression of KLF4, treatment with baicalein can stably express MARCH5 through promoting the expression of KLF4 after exposure to H_2_O_2_.

Drp1 is a key dynamin‐related GTPase and plays essential role in mitochondrial fusion and fission, then participates in autophagy or apoptosis.^13^, ^29^ The expression level of Drp1 was markedly increased after exposure to H_2_O_2_ (Figure 6A,C)_,_ and treatment with baicalein can signally reduce the expression level of Drp1 (Figure 6D,F). There is remarkable negative correlation between Drp1 and MARCH5 (Figures 1A and 5A). Drp1 can be ubiquitylated by ubiquitin ligases and then degraded.^9^ In order to probe wether MARCH5 can ubiquitylate Drp1, we performed the immunoprecipitation assay and the result showed in Figure 6G. In the MARCH5 knockout group, the expression level of Drp1 was significantly higher, but the ubiquitination level was the lowest in the H_2_O_2_ treated three groups. In the MARCH5 overexpression group, the expression level of Drp1 was remarkably reduced, but the ubiquitination level was the highest in the H_2_O_2_ treated three groups. In baicalein treatment group, the expression level of Drp1 was significantly decreased and the ubiquitination level was higher than other groups (Figure 6H). Altogether, we investigated that MARCH5 played crucial role in the ubiquitination of Drp1, MARCH5 could decrease the expression level of Drp1 via promoting it ubiquitination.

### 3.6 Baicalein attenuates myocardial ischaemia reperfusion injury

**Figure 7:**
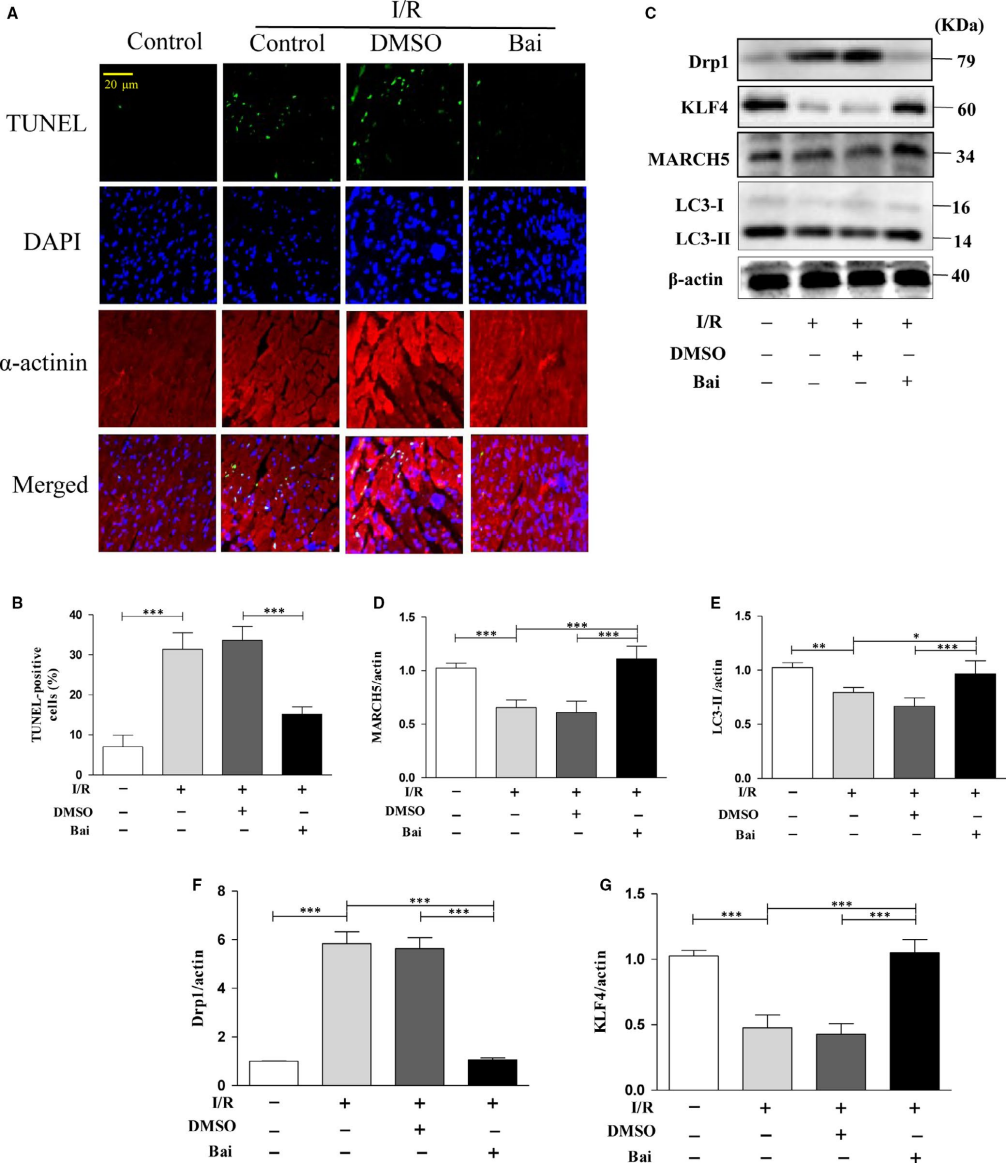
Baicalein attenuate myocardial I/R injury. Mice were undergoing I/R, and the myocardial tissue sections were used for evaluation cells apoptosis (A) by TUNEL assay. (B) shown the percentage of apoptotic cells. Mice were undergoing I/R, and the myocardial tissue was used for assessment proteins expression level by Western blotting (C) and densitometry (D–G). All of the data were expressed as the mean ± SEM of three independent experiments. *P < .05, **P < .01, ***P < .001

The oxidative stress induced by superoxide production during I/R is one of the main causes of cardiomyocytes death.^30^, ^31^ To understand whether baicalein can protect dells under oxidative stress in vivo, we further established I/R model in C57 mice. Our results revealed that the intervention of baicalein resulted in a reduction in apoptosis (Figure 7A,B). We also determined the MARCH5, LC3‐II, Drp1 and KLF4 expression level in cardiac tissue, the results shown that under baicalein intervention, the expression level of MARCH5 (Figure 7C,D), LC3‐II (Figure 7C,E) and KLF4 (Figure 7C,G) were obviously higher than the control group, while the expression level of Drp1 (Figure 7C,F) was significantly decreased than the related control group. Taken together, these data suggested that baicalein can reverse the injury of cardiac tissue caused by I/R via KLF4‐MARCH5‐Drp1 pathway.

## 4 DISCUSSION

Mitochondria have an important role for the cellular homoeostasis. Mitochondria are highly dynamic organelles that constantly undergo fusion and fission to correct the shape and distribution of the mitochondrial network according to energy requirements. The homoeostasis of mitochondrial is indispensable for the maintenance of normal physiological activity of cells.^1^, ^22^ Here we demonstrate the pivotal role of MARCH5 in affecting mitochondrial dynamics via regulating Drp1 ubiquitin. MARCH5 is down‐regulated under the oxidative stress in vitro and in vivo, it can inhibit mitochondrial fission, promote mitophagy and reduce apoptosis. Our results provide novel mechanism demonstrating the importance of MARCH5 on improving the survival of cardiomyocytes by maintaining mitochondrial homoeostasis.

Damaged mitochondria removed by mitophagy is an essential process in mitochondrial quality control.^15^ In this study, we found that oxidative stress can inhibit mitophagy, which led to the accumulation the accumulation of damaged mitochondria, and then induce cells apoptosis.

Recent research reported that MARCH5 plays an anti‐apoptotic role against ER stress.^11^ In this study, we identify MARCH5 as a key factor to maintain mitochondrial homoeostasis and mitophagy. Loss intracellular MARCH5 enhanced mitochondrial fission, inhibited mitophagy and then induced cells apoptosis (Figure 3).

Baicalein as a bioactive component present in Chinese herbal medicine, possesses a wide range of pharmacological activities with excellent oxidant scavenging capability. It has been reported to exert a cardioprotective effect by effective oxidant scavenging.^32^ Baicalein can protect cardiomyocytes by reduction oxidative stress, myocardial inflammatory responses and apoptosis in LPS‐induced sepsis.^33^ It also can attenuate LPS‐induced TNF‐a, IL‐6, NO and iNOS expression in neonatal rat cardiomyocytes.^34^ Here we suggested a novel point, baicalein conspicuous stabilized MARCH5 expression by promoting the expression of KLF4, and then promoted mitochondrial fusion, stabilized mitophagy and reduce apoptosis, this properties of baicalein could significantly attenuate oxidative damage caused by H_2_O_2_ or I/R.

In summary, we firstly reported a new cardioprotective pathway: KLF4‐MARCH5‐Drp1. Our present study reveals that MARCH5 participates in the machinery of mitochondrial dynamics and affects mitophagy. Moreover, we demonstrated that baicalein can exert its biological functions by stabilizing MARCH5 expression. Thus, modulation of MARCH5 may represent a novel strategy for the treatment of heart disease, we also provide new insight into understanding the cardioprotective property of baicalein. This finding may provide theoretical foundation for the internationalization of traditional Chinese medicine.

## CONFLICT OF INTEREST

No conflicts of interest exist.

## AUTHOR CONTRIBUTIONS

Qi Li, Yi An,Yufang Gao generated the idea; Qi Li, Dandan Xiao and Yu Wang performed the experiment; Lin Zhao and Qi Li analysed the data; Zhongjie Yu and Qi Li prepared and edited the manuscript.

## Data Availability

The data that support the findings of this study are available from the corresponding author upon reasonable request.

## References

[1] Wang K , Long B , Zhou LY , et al. CARL lncRNA inhibits anoxia‐induced mitochondrial fission and apoptosis in cardiomyocytes by impairing miR‐539‐dependent PHB2 downregulation. Nature Commun. 2014;5:3596. 24710105 10.1038/ncomms4596

[2] Song M , Mihara K , Chen Y , Scorrano L 2nd , Dorn GW . Mitochondrial fission and fusion factors reciprocally orchestrate mitophagic culling in mouse hearts and cultured fibroblasts. Cell Metab. 2015;21(2):273‐286. 25600785 10.1016/j.cmet.2014.12.011 PMC4318753

[3] Song M , Gong G , Burelle Y , et al. Interdependence of Parkin‐mediated mitophagy and mitochondrial fission in adult mouse hearts. Circ Res. 2015;117(4):346‐351. 26038571 10.1161/CIRCRESAHA.117.306859 PMC4522211

[4] Reddy PH . Inhibitors of mitochondrial fission as a therapeutic strategy for diseases with oxidative stress and mitochondrial dysfunction. J Alzheimer's Dis. 2014;40(2):245‐256. 24413616 10.3233/JAD-132060 PMC3972337

[5] Tong M , Saito T , Zhai P , et al. Mitophagy is essential for maintaining cardiac function during high fat diet‐induced diabetic cardiomyopathy. Circ Res. 2019;124(9):1360‐1371. 30786833 10.1161/CIRCRESAHA.118.314607 PMC6483841

[6] Zhang Y , Yao Y , Qiu X , et al. Listeria hijacks host mitophagy through a novel mitophagy receptor to evade killing. Nat Immunol. 2019;20(4):433‐446. 30804553 10.1038/s41590-019-0324-2

[7] Zhang Y , Wang Y , Xu J , et al. Melatonin attenuates myocardial ischemia‐reperfusion injury via improving mitochondrial fusion/mitophagy and activating the AMPK‐OPA1 signaling pathways. J Pineal Res. 2019;66(2):e12542. 30516280 10.1111/jpi.12542

[8] Nathan JA , Lehner PJ . The trafficking and regulation of membrane receptors by the RING‐CH ubiquitin E3 ligases. Exp Cell Res. 2009;315(9):1593‐1600. 19013150 10.1016/j.yexcr.2008.10.026

[9] Yonashiro R , Ishido S , Kyo S , et al. A novel mitochondrial ubiquitin ligase plays a critical role in mitochondrial dynamics. EMBO J. 2006;25(15):3618‐3626. 16874301 10.1038/sj.emboj.7601249 PMC1538564

[10] Zhang C , Shi Z , Zhang L , et al. Appoptosin interacts with mitochondrial outer‐membrane fusion proteins and regulates mitochondrial morphology. J Cell Sci. 2016;29(5):994‐1002. 10.1242/jcs.176792 PMC4813315 26813789

[11] Takeda K , Nagashima S , Shiiba I , et al. MITOL prevents ER stress‐induced apoptosis by IRE1alpha ubiquitylation at ER‐mitochondria contact sites. EMBO J. 2019;38:e100999. 31368599 10.15252/embj.2018100999 PMC6669929

[12] Xu S , Cherok E , Das S , et al. Mitochondrial E3 ubiquitin ligase MARCH5 controls mitochondrial fission and cell sensitivity to stress‐induced apoptosis through regulation of MiD49 protein. Mol Biol Cell. 2016;27(2):349‐359. 26564796 10.1091/mbc.E15-09-0678 PMC4713136

[13] Nakamura N , Kimura Y , Tokuda M , Honda S , Hirose S . MARCH‐V is a novel mitofusin 2‐ and Drp1‐binding protein able to change mitochondrial morphology. EMBO Rep. 2006;7(10):1019‐1022. 16936636 10.1038/sj.embor.7400790 PMC1618377

[14] Tang H , Peng S , Dong Y , et al. MARCH5 overexpression contributes to tumor growth and metastasis and associates with poor survival in breast cancer. Cancer Manag Res. 2019;11:201‐215. 30636894 10.2147/CMAR.S190694 PMC6307674

[15] Chen Z , Siraj S , Liu L , Chen Q . MARCH5‐FUNDC1 axis fine‐tunes hypoxia‐induced mitophagy. Autophagy. 2017;13(7):1244‐1245. 28486049 10.1080/15548627.2017.1310789 PMC5529064

[16] Nagai T , Yamada H , Otsuka Y . Inhibition of mouse liver sialidase by the root of Scutellaria baicalensis. Planta Med. 1989;55(1):27‐29. 2717686 10.1055/s-2006-961769

[17] Li‐Weber M . New therapeutic aspects of flavones: the anticancer properties of Scutellaria and its main active constituents Wogonin, Baicalein and Baicalin. Cancer Treat Rev. 2009;35(1):57‐68. 19004559 10.1016/j.ctrv.2008.09.005

[18] Choi EO , Park C , Hwang HJ , et al. Baicalein induces apoptosis via ROS‐dependent activation of caspases in human bladder cancer 5637 cells. Int J Oncol. 2016;49(3):1009‐1018. 27571890 10.3892/ijo.2016.3606

[19] Fan GW , Zhang Y , Jiang X , et al. Anti‐inflammatory activity of baicalein in LPS‐stimulated RAW264.7 macrophages via estrogen receptor and NF‐kappaB‐dependent pathways. Inflammation. 2013;36(6):1584‐1591. 23892998 10.1007/s10753-013-9703-2

[20] Van Leyen K , Kim HY , Lee SR , Jin G , Arai K , Lo EH . Baicalein and 12/15‐lipoxygenase in the ischemic brain. Stroke. 2006;37(12):3014‐3018. 17053180 10.1161/01.STR.0000249004.25444.a5

[21] Liau PR , Wu MS , Lee CK . Inhibitory effects of *Scutellaria baicalensis* root extract on linoleic acid hydroperoxide‐induced lung mitochondrial lipid peroxidation and antioxidant activities. Molecules. 2020;24(11):E2143. 10.3390/molecules24112143 PMC6600317 31174346

[22] Wang K , Long B , Jiao JQ , et al. miR‐484 regulates mitochondrial network through targeting Fis1. Nat Commun. 2012;3:781. 22510686 10.1038/ncomms1770

[23] Li PF , Li J , Müller EC , Otto A , Dietz R , von Harsdorf R . Phosphorylation by protein kinase CK2: a signaling switch for the caspase‐inhibiting protein ARC. Mol Cell. 2002;10(2):247‐258. 12191471 10.1016/s1097-2765(02)00600-7

[24] Hariharan N , Zhai P , Sadoshima J . Oxidative stress stimulates autophagic flux during ischemia/reperfusion. Antioxid Redox Signal. 2011;14(11):2179‐2190. 20812860 10.1089/ars.2010.3488 PMC3085947

[25] Marshall RS , Hua Z , Mali S , McLoughlin F , Vierstra RD . ATG8‐binding UIM proteins define a new class of autophagy adaptors and receptors. Cell. 2019;177(3):766‐781.e24. 30955882 10.1016/j.cell.2019.02.009 PMC6810650

[26] Hariharan N , Maejima Y , Nakae J , Paik J , Depinho RA , Sadoshima J . Deacetylation of FoxO by Sirt1 plays an essential role in mediating starvation‐induced autophagy in cardiac myocytes. Circ Res. 2010;107(12):1470‐1482. 20947830 10.1161/CIRCRESAHA.110.227371 PMC3011986

[27] Jiang TX , Zou JB , Zhu QQ , et al. SIP/CacyBP promotes autophagy by regulating levels of BRUCE/Apollon, which stimulates LC3‐I degradation. Proc Natl Acad Sci USA. 2019;116(27):13404‐13413. 31213539 10.1073/pnas.1901039116 PMC6613085

[28] Gu H , Li Q , Huang S , et al. Mitochondrial E3 ligase March5 maintains stemness of mouse ES cells via suppression of ERK signalling. Nat Commun. 2015;6:7112. 26033541 10.1038/ncomms8112 PMC4458872

[29] Karbowski M , Neutzner A , Youle RJ . The mitochondrial E3 ubiquitin ligase MARCH5 is required for Drp1 dependent mitochondrial division. The J Cell Biol. 2007;178(1):71‐84. 17606867 10.1083/jcb.200611064 PMC2064424

[30] Zhang T , Zhang Y , Cui M , et al. CaMKII is a RIP3 substrate mediating ischemia‐ and oxidative stress‐induced myocardial necroptosis. Nat Med. 2016;22(2):175‐182. 26726877 10.1038/nm.4017

[31] Brand MD , Goncalves RL , Orr AL , et al. Suppressors of superoxide‐H2O2 production at site IQ of mitochondrial complex I protect against stem cell hyperplasia and ischemia‐reperfusion injury. Cell Metab. 2016;24(4):582‐592. 27667666 10.1016/j.cmet.2016.08.012 PMC5061631

[32] Li J , Chang WT , Li CQ , et al. Baicalein preventive treatment confers optimal cardioprotection by PTEN/Akt/NO activation. Am J Chinese Med. 2017;45(5):987‐1001. 10.1142/S0192415X17500525 28760044

[33] Lee YM , Cheng PY , Chim LS , et al. Baicalein, an active component of *Scutellaria baicalensis* Georgi, improves cardiac contractile function in endotoxaemic rats via induction of heme oxygenase‐1 and suppression of inflammatory responses. J Ethnopharmacol. 2011;135(1):179‐185. 21396999 10.1016/j.jep.2011.03.009

[34] Chen HM , Liou SF , Hsu JH , et al. Baicalein inhibits HMGB1 release and MMP‐2/‐9 expression in lipopolysaccharide‐induced cardiac hypertrophy. Am J Chinese Med. 2014;42(4):785‐797. 10.1142/S0192415X14500505 25004875

